# Renal infarction in a COVID-19 patient

**DOI:** 10.11604/pamj.2020.37.182.26187

**Published:** 2020-10-27

**Authors:** Guglielmo Mantica, Aldo Franco De Rose

**Affiliations:** 1 Department of Urology, Policlinico San Martino Hospital, University of Genova, Genoa, Italy

**Keywords:** Renal infarction, COVID-19, kidney infarction

## Image in medicine

**Figure 1:**
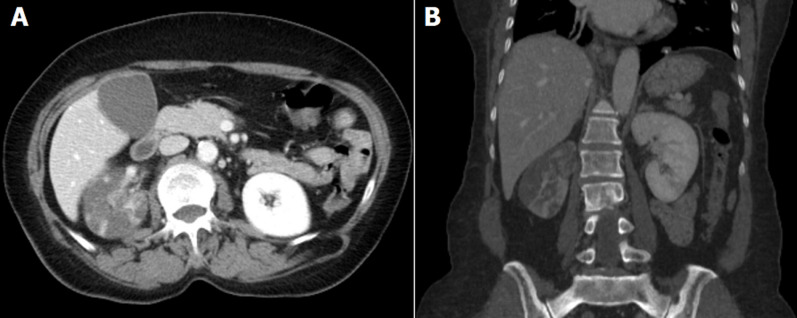
A) axial frame of the arterial phase of contrasted CT-scan in which is clearly visible the hypoperfusion of the right kidney compared to the left; B) coronal image of the abdomen in which is visible the hypoperfusion of the right kidney, especially in the upper pole

A 67-year-old female patient, who underwent a recent right lobectomy for pT2a N2 lung adenocarcinoma and adjuvant chemotherapy with Vinorelbine and Cisplatin, was admitted in April 2020 to the emergency department for nausea and abdominal pain. The clinical evaluation revealed a slightly positive right costovertebral angle tenderness and signs suspicious of right lower leg deep vein thrombosis. The blood tests revealed a white cell count of 26.07 X 10^9^/L; hemoglobin 13.8 g/dl; INR 1.17; C-reactive protein 170 mg/L and creatinine 1.0 mg/dl. The Figure 1 (A) shows an axial frame of the arterial phase of contrasted computed tomography (CT)-scan in which is clearly visible the hypoperfusion of the right kidney compared to the left. Only few sporadic areas of the right parenchyma pick up contrast, differently from the left kidney parenchyma which appears totally contrasted. Similarly, Figure 1 (B) shows a coronal image of the abdomen in which is visible the hypoperfusion of the right kidney, especially in the upper pole. The patient was started on fondaparinux 5 mg/day and Piperacillin/Tazobactam 4.5 g x 3/day. Few days after the admission the patient developed fever (38.9°C), dyspnea (Sp02 <90%) and tested positive for SARS-CoV-2. She died due to COVID-19. Although respiratory failure is the main characteristic of COVID-19, some authors indicate an increased risk for acute kidney injury (AKI). The pathophysiologic mechanisms leading to AKI in patients affected by SARS-CoV-2 are not completely clear but may include direct cytopathic effects of the virus on kidney tubular and endothelial cells or indirect damage caused by virus-induced cytokine release. Furthermore, thromboembolic events in COVID-19 patients are proven. Data suggest that the differential diagnosis of acute kidney injury in patients with COVID-19 infection should include kidney infarction. In this era of fear, patients and doctors should be aware of the fact that medical consultation should not be avoided or delayed in the absence of respiratory symptoms.

